# AMPK activation enhances the anti-atherogenic effects of high density lipoproteins in apoE^−/−^ mice

**DOI:** 10.1194/jlr.M073270

**Published:** 2017-06-13

**Authors:** Ang Ma, Jing Wang, Liu Yang, Yuanyuan An, Haibo Zhu

**Affiliations:** State Key Laboratory for Bioactive Substances and Functions of Natural Medicines, Beijing Key Laboratory of New Drug Mechanisms and Pharmacological Evaluation Study, and Institute of Materia Medica, Chinese Academy of Medical Sciences and Peking Union Medical College, Beijing, People’s Republic of China

**Keywords:** adenosine monophosphate-activated protein kinase, atherosclerosis, reverse cholesterol transport, apolipoprotein E deficient

## Abstract

HDL plays crucial roles at multiple stages of the pathogenesis of atherosclerosis. AMP-activated protein kinase (AMPK) is a therapeutic candidate for the treatment of cardiovascular disease. However, the effect of AMPK activation on HDL functionality has not been established in vivo. We assessed the effects of pharmacological AMPK activation using A-769662, AICAR, metformin, and IMM-H007 on the atheroprotective functions of HDL in apoE-deficient (apoE^−/−^) mice fed with a high-fat diet. After administration, there were no changes in serum lipid levels among the groups. However, mice treated with AMPK activators showed significantly enhanced reverse cholesterol transport in vivo and in vitro. AMPK activation also increased the expression of ABCA1 and ABCG1 in macrophages and scavenger receptor class B type I and LCAT in the liver. HDL from AMPK activation mice exhibited lower HDL inflammatory index and myeloperoxidase activity and higher paraoxonase 1 activity than HDL from untreated mice, implying superior antioxidant and anti-inflammatory capacities. Pharmacological AMPK activation also induced polarization of macrophages to the M2 state and reduced plasma lipid peroxidation, inflammatory cytokine production, and atherosclerotic plaque formation in apoE^−/−^ mice. These observations suggest that pharmacological AMPK activation enhances the anti-atherogenic properties of HDL in vivo. This likely represents a key mechanism by which AMPK activation attenuates atherosclerosis.

Epidemiological studies consistently show an inverse relationship between HDL cholesterol (HDL-C) levels and cardiovascular disease risk, suggesting a plausible therapeutic strategy of raising HDL-C to protect against atherosclerosis (1). However, a number of recent studies demonstrated that the level of circulating HDL-C alone represents an inadequate indicator of therapeutic efficacy (2). Instead, the functionality of HDL is more informative than circulating HDL-C levels in the evaluation of therapeutic approaches for atherosclerosis (3). HDL has multiple atheroprotective effects. It facilitates the efflux and transport of excess cholesterol from arterial macrophages to the liver/biliary tract, following the reverse cholesterol transport (RCT) pathway, which is thought to be the most crucial mechanism by which HDL protects against atherosclerosis (4, 5). RCT relies on specific interactions between HDL particles and both macrophages and hepatocytes. Of the proteins that mediate RCT, some contribute to cholesterol efflux, including ABCA1, ABCG1, and scavenger receptor class B type I (SR-BI), whereas others are involved in the maturation and hepatic uptake of HDL, such as LCAT and SR-BI (4).

HDL also has a number of potentially anti-atherogenic effects independent of RCT, including its antioxidant, anti-inflammatory, and endothelial cell maintenance effects, but the relative role of these activities in mediating HDL’s protective effect has not been directly addressed (2). The HDL inflammatory index (HII), which quantifies the pro- or anti-inflammatory properties of HDL, is a useful marker of susceptibility to atherogenesis (6). Furthermore, myeloperoxidase (MPO) and paraoxonase 1 (PON1) are HDL-associated proteins that bind to HDL and are mechanistically linked to inflammation, oxidative stress, and atherosclerosis (7). Moreover, HDL may become dysfunctional during chronic inflammation and oxidative stress. Thus, various approaches aimed at enhancing HDL function have gained favor as potentially more effective anti-atherosclerotic therapies.

AMP-activated protein kinase (AMPK), a phylogenetically conserved serine/threonine kinase that is widely recognized as a key regulator of lipid and energy metabolism, is emerging as a promising molecular target for the treatment of cardiovascular disease (8, 9). Accumulating evidence demonstrates that AMPK exerts multiple anti-atherosclerotic effects by promoting vascular health (10). In addition, the activation of AMPK can restore cholesterol homeostasis in macrophages through the suppression of foam cell formation during the early stages of atherosclerosis (11, 12). Recent findings also suggest that AMPK activation reduces inflammation, which is one of the main causes of atherosclerosis by promoting macrophage transition from a pro-inflammatory (M1) to an anti-inflammatory (M2) phenotype (13, 14). However, the effects of pharmacological activation of AMPK on the anti-atherogenic properties of HDL have not been investigated in a suitable in vivo model.

AMPK exists as a heterotrimer consisting of a catalytic α subunit and regulatory β and γ subunits (15). A number of compounds have been identified that activate AMPK in different ways. The anti-diabetic drug, metformin, activates AMPK indirectly, while the widely used compound, 5-aminoimidazole-4-carboxamide ribonucleotide (AICAR), activates AMPK after its intracellular conversion to the AMP analog, ZMP, which binds to the γ subunit. Another direct activator, A-769662, increases AMPK activity by binding to the β1 subunit (16, 17), while our previous work showed that triacetyl-3-hydroxyphenyladenosine (IMM-H007) activates AMPK by directly binding to the γ subunit (11, 18). Although all of these activities have atheroprotective effects (10–12), the importance of AMPK and the mechanism of AMPK activation for HDL function and atheroprotection are not fully understood.

In the current study, we systematically assessed the impact of activating AMPK with four different agonists on the properties of HDL in a murine model of atherosclerosis. We examined the effect of these agonists on HDL-mediated RCT and HDL-mediated anti-inflammatory and antioxidant actions. We show that all these agonists improve the function of HDL and reduce atherosclerosis in mice. These findings further support the potential of AMPK as a drug target for the treatment of atherosclerosis-related cardiovascular disease.

## MATERIALS AND METHODS

### Reagents

A-769662 was provided by Santa Cruz. AICAR and 8-Br-cAMP were purchased from Sigma and metformin from Beyotime Institute of Biotechnology. IMM-H007 was provided by the Institute of Material Medica, Chinese Academy of Medical Sciences and Peking Union Medical College (99.86% purity by HPLC). Acetylated (ac)LDL was obtained from Peking Union-Biology Co. Ltd. The [1,2-^3^H(N)]cholesterol and scintillation cocktails were purchased from PerkinElmer Life Sciences. The 1-palmitoyl-2-arachidonyl-*sn*-glycero-3-phosphorylcholine (PAPC) and hydroperoxyoctadecadienoic acid (HPODE) were purchased from Avanti Polar Lipids; dichlorofluorescein diacetate was purchased from Molecular Probes; and 1-palmitoyl-2-(5,6-epoxyisoprostaneE (2))-*sn*-glycero-3-phosphocholine was prepared from PAPC as described previously (19, 20).

### Animals

Male apoE-deficient (apoE^−/−^) mice on a C57BL/6 background, weighing 18–20 g, were purchased from Vital River Laboratory Animal Technology Co. Ltd. (Beijing, China). Mice were fed a high-fat diet (10% lard, 1.2% cholesterol w/w) in a SPF laboratory animal facility at 25°C under a 12 h light-dark cycle. They were treated with A-769662 (30 mg/kg, ip), AICAR (200 mg/kg, ip), metformin (260 mg/kg, by gavage), IMM-H007 (200 mg/kg, by gavage), or sodium carboxymethylcellulose (control group) once a day for 10 weeks. The doses of AICAR and IMM-H007 were selected based on our previous studies (11, 21); the dose of A-769662 was selected based on a previous report (16), while the dose of metformin was calculated based on body surface area equivalent to therapeutic human dosing (2 g/day). Furthermore, the previous studies have shown that the doses of these activators all have satisfactory atheroprotective effects. All procedures were performed in accordance with the regulations of the Institutional Animal Care and Use Committee of the Institute of Materia Medica, Chinese Academy of Medical Sciences, and Peking Union Medical College (Beijing, China).

### Cell preparations and cultures

Murine J774A.1 macrophages (Cell Culture Center of Peking Union Medical College, China) were cultured in DMEM plus 10% FBS, penicillin, and streptomycin. Mouse peritoneal macrophages were collected from mice 3 days after intraperitoneal injection with 1 ml of 6% starch broth, as described previously (22). After the mice were anesthetized, approximately 10 ml of ice-cold PBS was injected into the peritoneal cavity of each mouse. The fluid was then carefully collected, centrifuged at 500 *g* for 5 min, and resuspended in RPMI 1640 with 10% FBS. Flasks were then placed in a 5% CO_2_-containing incubator for 4 h for the cells to adhere, followed by three washes with PBS to remove nonadherent cells.

### Serum analyses

After 10 weeks of treatment, blood samples were obtained from the retro-orbital sinus of mice (n = 10/group). The concentrations of serum total cholesterol (TC) and triglyceride (TG) were directly measured by using commercial kits from SEKISUI Company, Japan. HDL-C levels were also measured by cholesterol enzymatic kit (SEKISUI Company) after precipitation of apoB-containing lipoproteins, as described before (23). LDL cholesterol (LDL-C) was calculated using the Friedewald formula (24) [LDL-C (mg/dl) = TC − HDL-C − TG/5]. Serum PON1 activity was measured as previously described (25). The serum activities of MPO, malondialdehyde (MDA), and superoxide dismutase (SOD) were measured using commercial kits (Nanjing Jiancheng Biochemistry, China). Serum inflammatory biomarkers [MDA, SOD, interleukin (IL)-12, p70, TNF-α, IFN-γ, monocyte chemoattractant protein-1 (MCP-1), IL-10, and IL-6] were quantified using the Cytometric Bead Array Mouse Inflammation Kit (BD Biosciences, San Jose, CA).

### Measurement of in vivo RCT

Experiments were carried out as described previously (12, 21, 26). apoE^−/−^ mice were fed a high-fat diet and administered with vehicle or AMPK activators for 2 weeks. J774 cells were loaded with 50 μg/ml acLDL and 5 μCi/ml ^3^H-cholesterol for 24 h in vitro and equilibrated in DMEM supplemented with 0.2% BSA overnight. Cells were washed and scraped into fresh DMEM/0.2% BSA, centrifuged at 1,200 *g* for 5 min, and resuspended in DMEM. The labeled J774 cells (4.5 × 10^6^ cells/mouse, 3 × 10^6^ cpm in 0.25 ml DMEM, n = 6/group) were injected into the peritoneal cavity of individually housed mice. Plasma samples were collected at 6, 24, and 48 h after injection, and 10 μl aliquots were counted in a scintillation counter. Feces were collected over the whole 48 h, and the liver was removed after euthanasia for lipid extraction. Mice continued to receive vehicle or AMPK activator during the 48 h RCT study. Radioactivity was determined in plasma, liver, and total feces by liquid scintillation counting. All ^3^H-tracer data are expressed as percentages of the cpm per mouse of the cpm of the initially injected ^3^H-tracer.

### In vitro cholesterol efflux experiment

Cholesterol efflux experiments were performed as previously described (21). Briefly, J774A.1 macrophages plated in 24 multi-well plates, were labeled with ^3^H-cholesterol (2 μCi/ml) and in the presence of 0.3 mM 8-Br-cAMP in DMEM plus 1% FBS for 24 h. After the labeling period, cells were washed and equilibrated overnight in medium with 0.2% BSA. Cholesterol efflux was performed for 4 h by the addition of medium plus 0.2% BSA with AMPK activator-treated apoB-depleted serum [polyethylene glycol (PEG)-HDL]. Radioactivity was measured in the medium and cell lysate, and efflux was calculated as percent radioactivity in the medium divided by total radioactivity in cells and medium (27).

### Western blot analysis

Liver and peritoneal macrophages were lysed in RIPA buffer containing a cocktail of protease and phosphatase inhibitors (Roche). Protein concentrations of all samples were measured using the BCA Protein Assay (MACGENE, China), and equal amounts of protein from each sample were separated by SDS-PAGE on 10% gels and transferred to PVDF membranes (Millipore). After blocking in TBST containing 5% BSA, membranes were incubated with primary antibodies targeting ABCA1 (1:1,000; Abcam), ABCG1 (1:1,000; Abcam), SR-BI (1:2,000; Abcam), LCAT (1:1,000; Abcam), liver X receptor (LXR)-α (1:500; Abcam), AMPK (1:1,000; Cell Signaling Technology), p-AMPK (1:1,000; Cell Signaling Technology), or β-actin (1:10,000; Abcam) at 4°C overnight. Membranes were subsequently incubated with HRP-conjugated goat anti-mouse or goat anti-rabbit secondary antibodies (ZSGB-BIO, China). The protein bands were visualized and quantified using a chemiluminescence method (ECL Plus Western blotting detection system; GE Healthcare UK Ltd.).

### Quantitative real-time PCR

To evaluate gene expression in liver or peritoneal macrophages, total RNA was extracted by TRIzol reagent (Invitrogen, Gaithersburg, MD) in accordance with manufacturer’s instructions and converted into cDNA by reverse transcriptase (TOYOBO, Japan). Real-time quantitative PCR (qPCR) was undertaken on an ABI Prism 7900 Fast real-time PCR system with SYBR Green detection. The relative levels of the mRNAs were calculated with β-actin mRNA as the invariant control. Relative transcript expression was determined using control sample as a calibrator and the ^ΔΔ^Ct method.

### Measurement of HII

HII was determined by using a cell-free assay that measured the ability of apoB-depleted serum to prevent the oxidation of PAPC by HPODE. apoB-containing lipoproteins were precipitated by adding 50 μl of serum to 50 μl of 20% PEG 6000 (pH 10), and the HDL-containing supernatant (PEG-HDL) was used in the assay. Dichlorofluorescein diacetate (2 mg/ml) was dissolved in fresh methanol and then incubated at room temperature, protected from light, for 30 min to release DCFH. PAPC and HPODE were prepared as previously described (28, 29), then 10 μl of PAPC (2.5 mg/ml), 10 μl of HPODE (0.1 mg/ml), and 25 μl of apoB-depleted serum were diluted in PBS to a final volume of 90 μl and incubated at 37°C in 96-well microtiter plates (Corning) for 4 h, with rotation. Ten microliters of DCFH solution (0.2 mg/ml) were then added to each well, mixed, and incubated for an additional 2 h at 37°C, with rotation. Fluorescence intensity was determined using a plate reader set at an excitation wavelength of 485 nm and an emission wavelength of 530 nm. Index values in the absence of HDL were normalized to 1.0. Values >1.0 after the addition of the test HDL indicated dysfunctional pro-inflammatory HDLs, while values <1.0 indicated normal anti-inflammatory HDLs.

### Flow cytometry

Mouse peritoneal macrophages were incubated with conjugated antibody against various cell surface markers, including CD206, CD11C, CD11b, and F4/80 (BD Biosciences). Cells were acquired on a Beckmen Gallios and analysis was performed with Flow Jo software (Tree Star, Inc., Ashland, OR).

### Statistical analysis

Data are expressed as mean ± SEM. Statistical significance was evaluated by Student’s *t*-test for differences between two groups, and one-way ANOVA with Tukey’s post hoc test was used for multiple groups. GraphPad Prism software version 6.0 was used for these analyses and *P* < 0.05 was considered statistically significant.

## RESULTS

### Effect of AMPK activation on serum lipid and body weight of apoE^−/−^mice

It has been established that increased plasma lipid levels can contribute to the initiation and progression of atherosclerosis (30). AMPK, as a regulator of glucose and lipid metabolism, has been reported to decrease both plasma glucose and TG levels in ob/ob mice when it is activated (16). To investigate whether AMPK activation could influence serum lipid in a mouse model of atherosclerosis, apoE^−/−^ mice fed a high-fat diet were treated with vehicle, A-769662 (30 mg/kg, ip), AICAR (200 mg/kg, ip), metformin (260 mg/kg, by gavage), or IMM-H007 (200 mg/kg, by gavage). The serum lipid-profile was determined after 10 weeks of treatment with AMPK activators. As shown in **Table 1**, the levels of TC, TG, LDL-C, and body weight were not altered significantly by AMPK activator treatment.

**TABLE 1.** Biochemical characteristics of apoE^−/−^ mice treated with or without AMPK activator

Variables	Control	A-769662	AICAR	Metformin	IMM-H007
TC	29.57 ± 1.37	25.46 ± 1.55	25.43 ± 1.50	28.03 ± 1.42	28.24 ± 2.12
LDL-C	25.27 ± 1.73	24.09 ± 1.67	24.17 ± 1.60	26.78 ± 1.46	26.49 ± 1.83
HDL-C	1.07 ± 0.15	1.10 ± 0.12	0.99 ± 0.15	1.02 ± 0.08	1.51 ± 0.30
TG	1.14 ± 0.12	1.33 ± 0.29	1.35 ± 0.27	1.14 ± 0.18	1.18 ± 0.10
BW	29.14 ± 0.74	28.19 ± 0.55	28.58 ± 0.61	27.69 ± 0.58	27.40 ± 0.57

TC, LDL-C, HDL-C, and TG are in millimoles per liter and BW is in grams. All values are presented as mean ± SEM (n = 10). BW, body weight.

### AMPK activation promoted RCT in vivo and in vitro

**Fig. 1.**
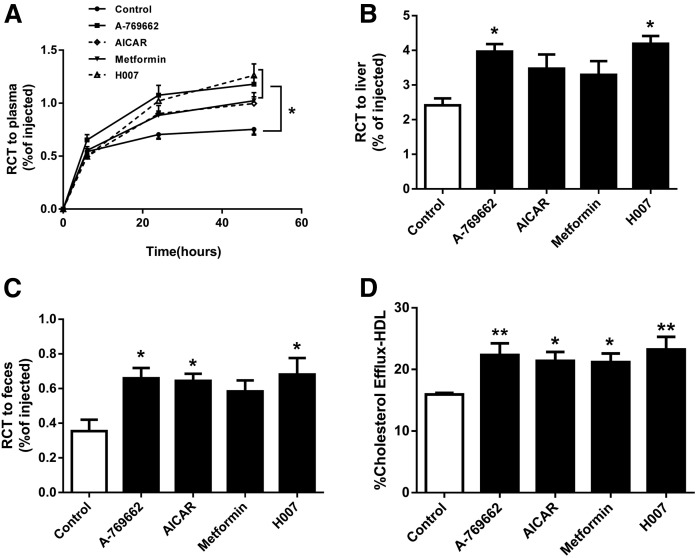
AMPK activation enhances RCT in vivo and vitro. ^3^H-cholesterol and acLDL-loaded J774 macrophages were injected into apoE^−/−^ mice fed a high-fat diet (containing 1.2% cholesterol and 10% lard) and treated with vehicle, A-769662 (30 mg/kg, ip), AICAR (200 mg/kg, ip), metformin (260 mg/kg, by gavage), or IMM-H007 (200 mg/kg, by gavage) for 2 weeks, followed by radioactive counting in the serum (A), liver (B), and feces (C). D: Cholesterol efflux from cAMP pretreated J774.A1 macrophages was measured by 4 h incubation of labeled cells to AMPK activator-treated PEG-HDL. Data are mean ± SEM for n = 6 per group. *P < 0.05, **P < 0.01 versus control.

One of the main anti-atherogenic functions of HDL is RCT. Previous studies demonstrated that in vivo RCT was suppressed in mice that received AMPK β^−/−^ macrophages versus WT controls (12). To investigate whether AMPK activation could enhance RCT in a mouse model of atherosclerosis, ^3^H-cholesterol-labeled J774 cells were injected into apoE^−/−^ mice after 14 days of treatment with AMPK activators or vehicle. The plasma levels of macrophage-derived ^3^H in mice treated with AMPK activators (A-769662, AICAR, metformin, or IMM-H007) were all significantly increased 48 h postinjection, by 66, 47, 42, and 73%, respectively, versus the control group (*P* < 0.05, *P* values are overall ANOVA effects) (**Fig. 1A**). Tritium counts 48 h after ^3^H-cholesterol-labeled J774 cells were injected into the peritoneal cavity of mice treated with AMPK activators (A-769662, AICAR, metformin, or IMM-H007) were substantially increased, by 64% (*P* < 0.05), 44%, 37%, and 74% (*P* < 0.05), respectively, in the liver (Fig. 1B), and 86% (*P* < 0.05), 82% (*P* < 0.05), 65%, and 92% (*P* < 0.05), respectively, in feces, versus the controls (Fig. 1C). To further understand whether the HDL from AMPK activator groups was functional or not, we performed cholesterol efflux assays on macrophages in the presence of AMPK activator-treated apoB-depleted serum (PEG-HDL). As shown in Fig. 1D, HDLs from AMPK activator-treated (A-769662, AICAR, metformin, or IMM-H007) groups were all significantly enhanced cholesterol efflux from J774 macrophages by 40% (*P* < 0.01), 34% (*P* < 0.05), 33% (*P* < 0.05), and 46% (*P* < 0.01), respectively, versus the control group. These observations suggest that pharmacological AMPK activation increases HDL-mediated RCT in vivo and in vitro.

### AMPK activation increases ABCA1 and ABCG1 protein expression in apoE^−/−^ peritoneal macrophages

**Fig. 2.**
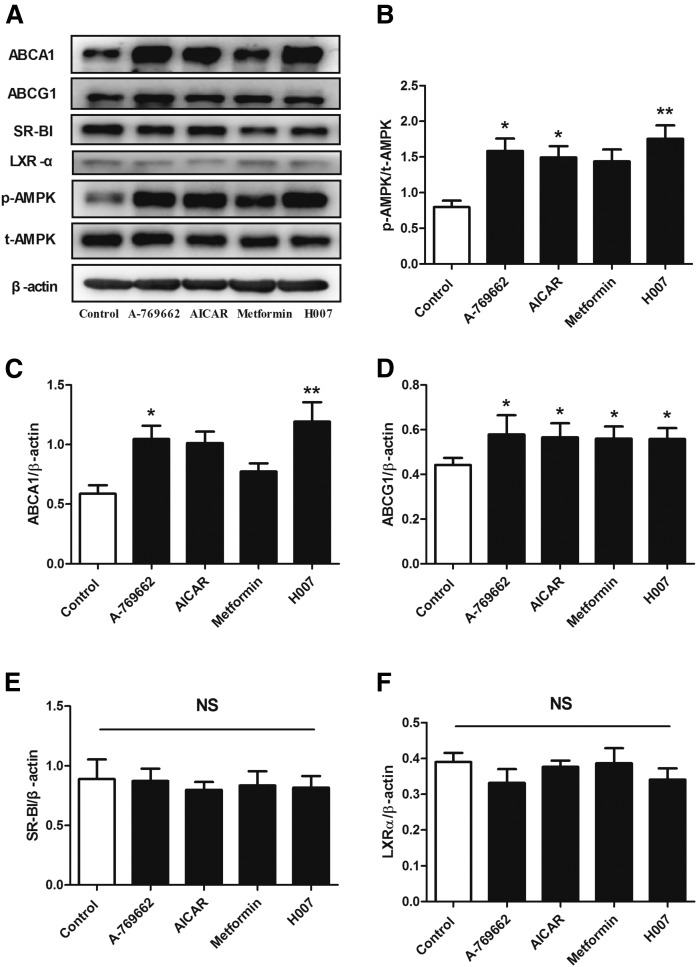
AMPK activation increases the protein expression of ABCA1 and ABCG1 in peritoneal macrophages of apoE^−/−^ mice. Peritoneal macrophages were prepared from apoE^−/−^ mice fed a high-fat diet (containing 1.2% cholesterol and 10% lard) and treated with vehicle, A-769662 (30 mg/kg, ip), AICAR (200 mg/kg, ip), metformin (260 mg/kg, by gavage), or IMM-H007 (200 mg/kg, by gavage) for 10 weeks. The protein expression of pT172-AMPK (A), AMPK (B), ABCA1 (C), ABCG1 (D), SR-BI (E), and LXR-α (F) in peritoneal macrophages was determined by Western blot. Data are mean ± SEM for n = 5 per group. *P < 0.05, **P < 0.01 versus control.

The ability of macrophages to generate efflux of cholesterol to extracellular acceptors is the first and critical step of RCT. To understand the mechanism whereby AMPK activation improves RCT, we initially evaluated the impact of chronic AMPK activation on the expression of cholesterol efflux proteins in macrophages (**Fig. 2A**). Seven-week-old apoE^−/−^ mice fed a high-fat diet were randomized to receive vehicle, A-769662, AICAR, metformin, or IMM-H007 for 10 weeks. We then isolated mouse peritoneal macrophages and measured AMPK activity and cholesterol efflux protein expression. Compared with no treatment, treatment with A-769662, AICAR, metformin, or IMM-H007 respectively increased phosphorylation of AMPK by 45% (*P* < 0.05), 34% (*P* < 0.05), 30%, and 50% (*P* < 0.01) in Thr-172 (Fig. 2B), which is a marker of AMPK activity (25). Furthermore, treatment with any of the AMPK activators increased or tended to increase the expression of ABCA1 and ABCG1 protein (Fig. 2C, D), but not SR-BI protein (Fig. 2E). However, pharmacological AMPK activation did not alter the expression of LXR-α (Fig. 2F), which is a key transcription factor that positively regulates ABCA1 and ABCG1 expression. These findings imply that AMPK activation regulated the expression of ABCA1 and ABCG1 in peritoneal macrophages not through increasing LXR-α expression, which may be through a posttranscriptional mechanism that stabilized the mRNA or protein (21, 31).

### AMPK activation induces SR-BI and LCAT expression in the liver of apoE^−/−^ mice

**Fig. 3.**
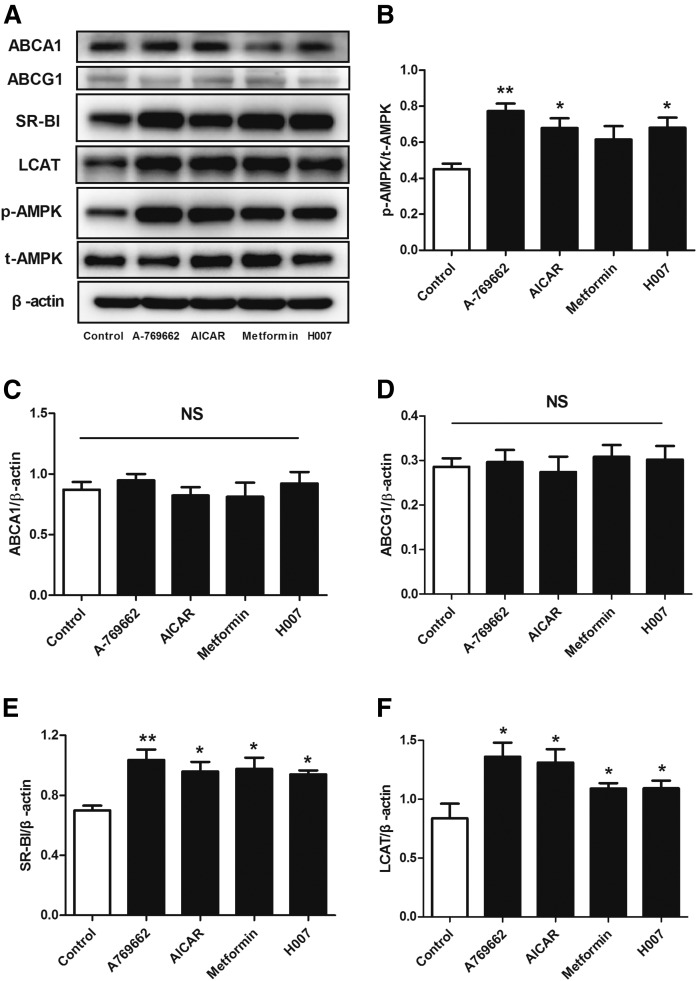
AMPK activation increases the protein expression of SR-BI and LCAT in the liver of apoE^−/−^ mice. The protein expression of pT172-AMPK (A), AMPK (B), ABCA1 (C), ABCG1 (D), SR-BI (E), and LCAT (F) in liver tissues was determined by Western blot. Data are mean ± SEM for n = 5–8 per group. *P < 0.05, **P < 0.01 versus control.

We showed that AMPK activation promotes overall RCT in vivo, but we also wished to investigate whether it could enhance cholesterol uptake by the liver. Hepatic HDL uptake requires a specific interaction between HDL particles and hepatocytes. Based on these facts, we measured the expression of proteins involved in RCT and AMPK activity in the livers of control and AMPK activator-treated mice (**Fig. 3**). The Thr-172 phosphorylation of AMPK, which reflects AMPK activity, was increased by 71% in the A-769662 group, by 51% in the AICAR group, by 36% in the metformin group, and by 51% in the IMM-H007 group, versus the control group (Fig. 3B). Additionally, AMPK activation did not affect ABCA1 and ABCG1 expression in the liver, but significantly increased the expression of SR-BI and LCAT (Fig. 3C–F).

### AMPK activation increases the mRNA expression of ABCA1 and ABCG1 in peritoneal macrophages and of SR-BI and LCAT in hepatocytes

**Fig. 4.**
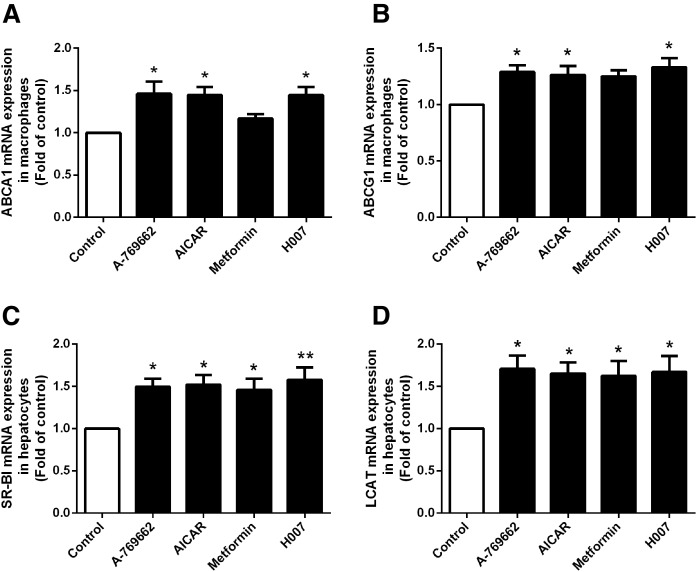
AMPK activation increases ABCA1 and ABCG1 mRNA expression in peritoneal macrophages and SR-BI and LCAT mRNA expression in the liver of apoE^−/−^ mice. The mRNA expression of cholesterol transport proteins in peritoneal macrophages and the liver of apoE^−/−^ mice was determined by real-time quantitative PCR (A–D, respectively). Data are mean ± SEM for n = 5 per group. *P < 0.05, **P < 0.01, versus control.

To further evaluate the regulation of proteins involved in cholesterol transport by AMPK activators, we assessed the effects of AMPK activation on the mRNA expression of ABCA1, ABCG1, SR-BI, and LCAT by quantitative RT-PCR. Peritoneal macrophages and hepatocytes were isolated from control and AMPK activator-treated groups. As shown in **Fig. 4**, treatment with any of the AMPK activators increased or tended to increase ABCA1 and ABCG1 mRNA in peritoneal macrophages (Fig. 4A, B) and SR-BI and LCAT mRNA in the liver versus the control (Fig. 4C, D), consistent with the effects of AMPK activation on the equivalent protein expression levels in apoE^−/−^ mice.

These results imply that AMPK activation promotes macrophage RCT by upregulating not only ABCA1 and ABCG1 expression in macrophages, but also SR-BI and LCAT expression in the liver in vivo.

### AMPK activation improves the anti-inflammatory and antioxidative properties of HDL in apoE^−/−^ mice

**Fig. 5.**
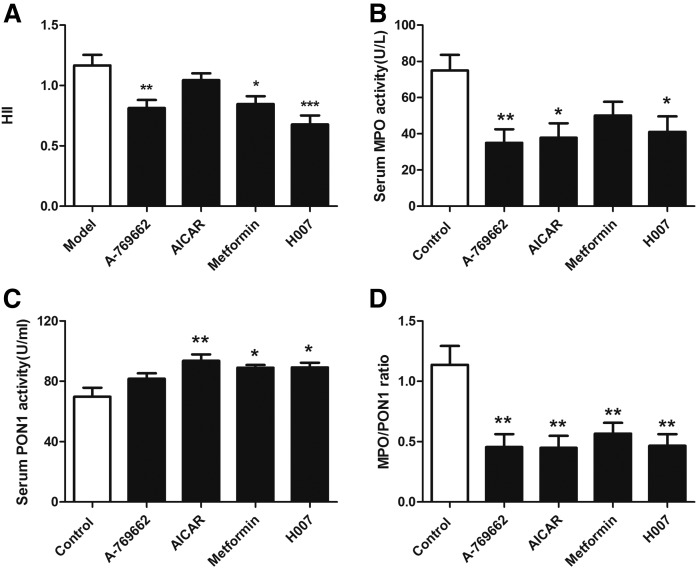
AMPK activation improves the anti-inflammatory and antioxidant effects of HDL in apoE^−/−^ mice. A: The HII. B: Serum activity of MPO. C: Serum activity of PON1. D: Ratio MPO/PON1. Data are mean ± SEM for n = 10 per group. *P < 0.05, **P < 0.01, ***P < 0.001 versus control.

HDL has a number of atheroprotective properties that are independent of RCT, such as its anti-inflammatory and antioxidative properties (2, 28). To assess the pro-inflammatory or anti-inflammatory properties of HDL in control and AMPK activation groups, HDL was isolated from each mouse and HII assays were performed (28). **Figure 5A** shows that HII values were improved by AMPK activation. The HII value above 1.0 measured in the control group indicates that the HDL is pro-inflammatory. Conversely, the HII values below 1.0 in most of the AMPK activator-treated groups denote anti-inflammatory effects of HDL.

Recent studies show that there is a specific interaction between MPO, apoAI, and PON1 on the surface of HDL and that the MPO/PON1 ratio could be a potential indicator of dysfunctional HDL and, thus, be used for risk stratification in coronary artery disease (32, 33). MPO selectively induces HDL oxidation, thereby impairing its atheroprotective functions. Conversely, PON1 has antioxidant properties and mediates some of the atheroprotective functions attributed to HDL (7, 33).We, therefore, evaluated the impact of AMPK activators on the activities of MPO and PON1 and their ratio. As expected, following treatment with AMPK activators, apoE^−/−^ mice showed diminished MPO activity and higher PON1 activity (Fig. 5B, C), meaning that the MPO/PON1 ratio was significantly decreased (Fig. 5D).

### AMPK activation induces the polarization of macrophages to M2 state and inhibits lipid peroxidation and inflammation in apoE^−/−^ mice

**Fig. 6.**
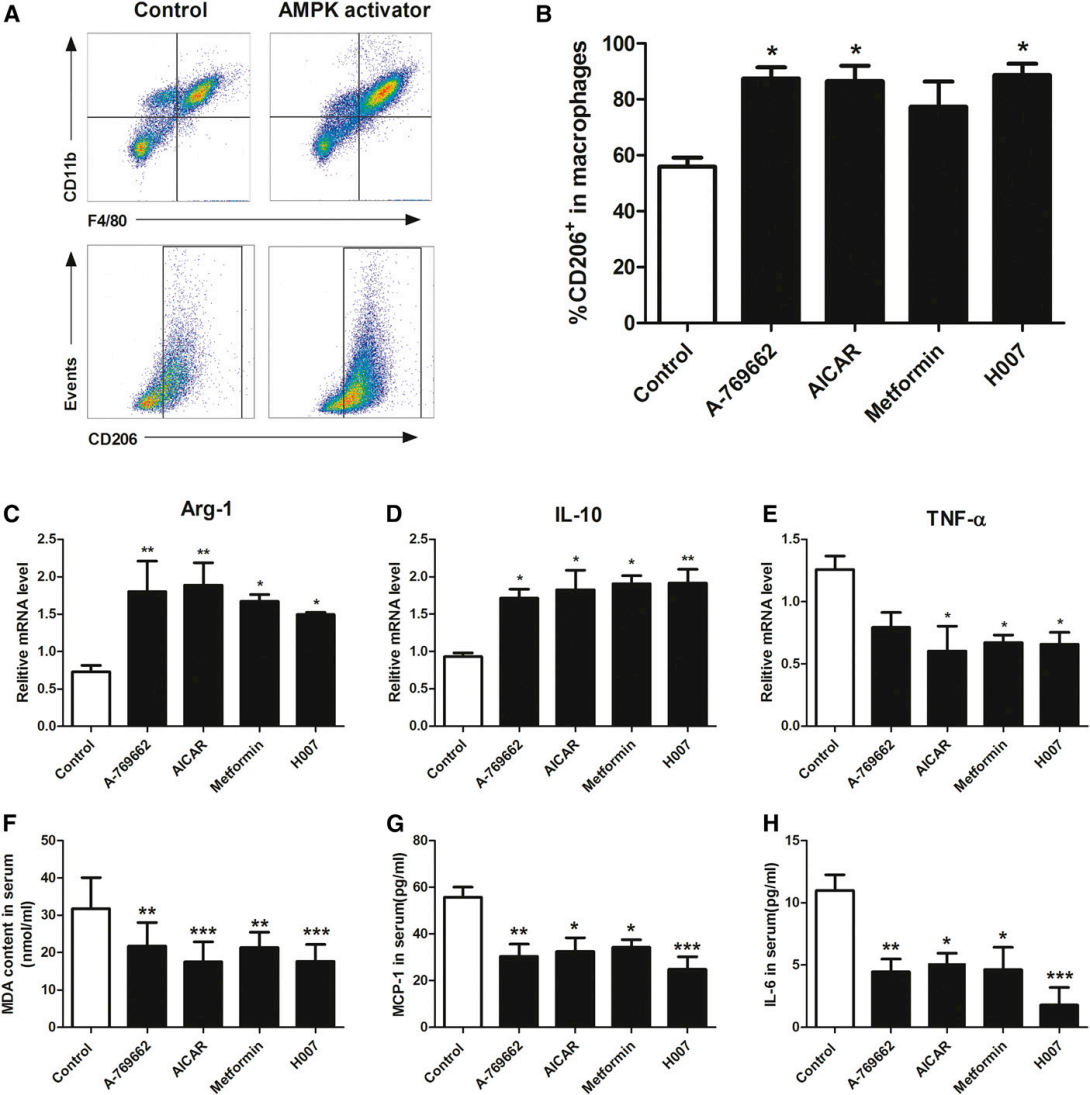
AMPK activation induces the polarization of macrophages to M2 state and inhibits lipid peroxidation and inflammation in apoE^−/−^ mice. Peritoneal macrophages were prepared from apoE^−/−^ mice fed a high-fat diet (containing 1.2% cholesterol and 10% lard) and treated with vehicle, A-769662 (30 mg/kg, ip), AICAR (200 mg/kg, ip), metformin (260 mg/kg, by gavage), or IMM-H007 (200 mg/kg, by gavage) for 10 weeks. A: Peritoneal macrophages were stained with monoclonal antibodies specific for F4/80, CD11b, and CD206. B: Percentage of CD206^+^ macrophages. Gene expression for arginase-1 (Arg-1) (C), IL-10 (D), and TNF-α (E) was assessed by real-time quantitative PCR. F: Serum concentration of MDA. G: Serum concentration of MCP-1. H: Serum concentration of IL-6. Data are mean ± SEM for n = 5–10 per group. *P < 0.05, **P < 0.01, ***P < 0.001 versus control.

Atherosclerosis is a pro-inflammatory disease that develops in the setting of hypercholesterolemia, in which HDL may be modified and lose its atheroprotective effects under inflammation and oxidative stress (34–36). AMPK is known to inhibit inflammation and production of reactive oxygen species by promoting macrophage M2 polarization (13). It is reported that M2-type macrophages express high levels of ABCA1 protein (37). To clarify whether AMPK activation could polarize macrophages to the M2 phenotype and reduce oxidative and inflammatory damage in this model and, therefore, play a role in the atheroprotective functions of HDL, we measured the phenotypic state of peritoneal macrophages and the serum levels of a range of oxidative and inflammatory biomarkers (MDA, SOD, IL-12, p70, TNF-α, IFN-γ, MCP-1, IL-10, and IL-6) in apoE^−/−^ mice. These analyses revealed that mice treated with AMPK activators had a significantly increased percentage of CD206-positive cells (M2) in peritoneal macrophages (**Fig. 6A, B**) and increased expression of M2 markers, arginase 1 and IL-10, and significantly decreased expression of the M1 marker, TNF-α, compared with controls (Fig. 6C–E). In addition, serum MDA, a lipid peroxidation product that can impair the atheroprotective function of HDL, was decreased significantly following AMPK activation (Fig. 6F). Serum MCP-1 and IL-6, both of which can reduce RCT, were decreased by these treatments (Fig. 6G, H). These data suggest that AMPK activation increases ABCA1/G1 expression in macrophages, but not the liver, and reduces inflammation and oxidative damage related to AMPK-induced polarization of macrophages to the M2 state, and therefore improves the anti-atherogenic function of HDL in apoE^−/−^ mice.

### Pharmacological AMPK activation suppresses the formation of atherosclerotic lesions in apoE^−/−^ mice

**Fig. 7.**
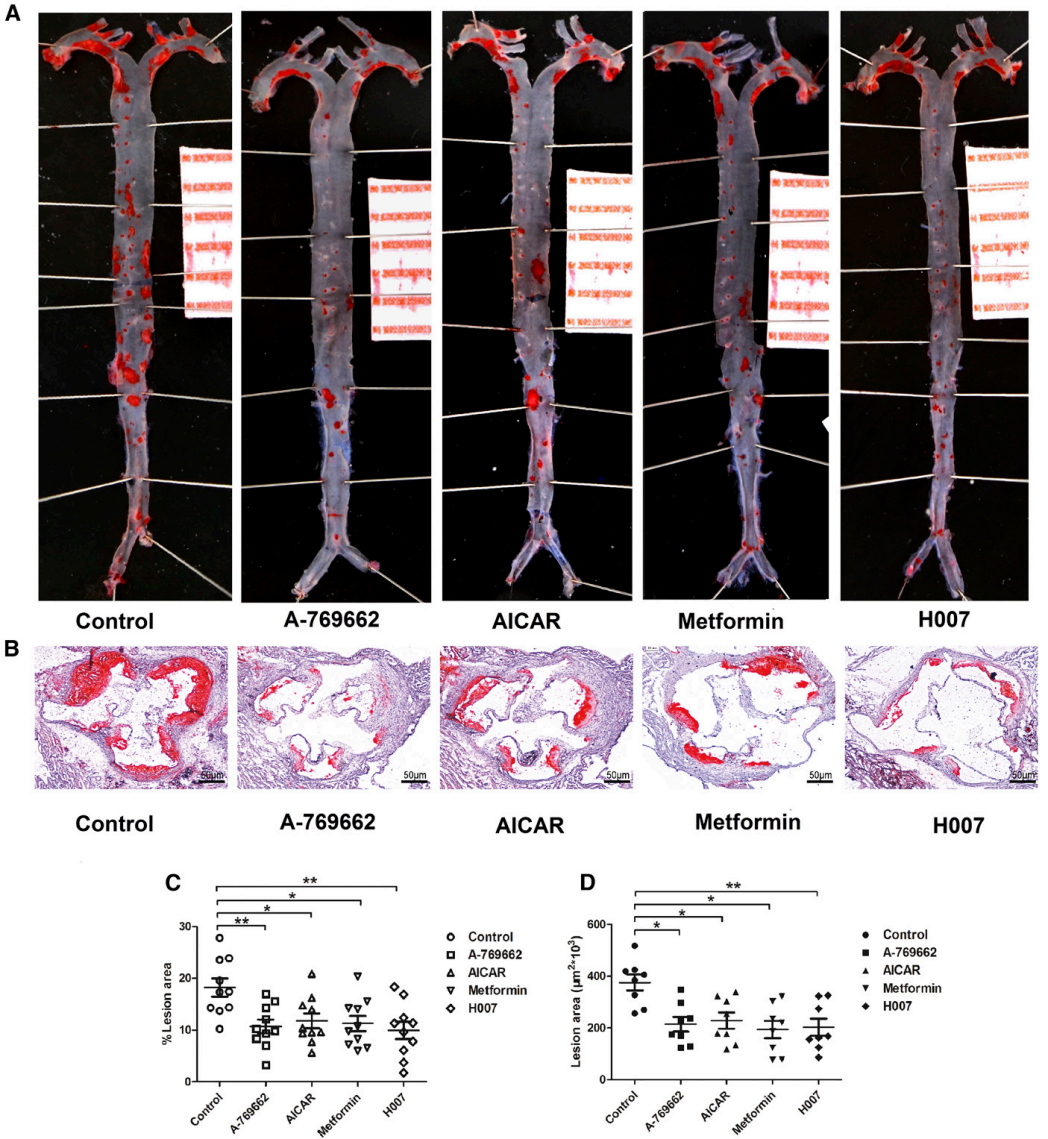
Pharmacological AMPK activation alleviates atherosclerotic plaque development in apoE^−/−^ mice. Representative images of dissected whole aortas (A) and cross-sections of aortic root (B) stained with oil red O are shown. Quantitative analyses of dissected whole aortas (C) and cross-sections of aortic root lesions (D) in apoE^−/−^ mice are shown to demonstrate the inhibitory effect of AMPK activation on atherosclerosis. Each data point represents an individual animal. The horizontal lines denote the mean for each treatment group. Values are expressed as mean ± SEM for n = 10 per group. *P < 0.05, **P < 0.01 versus control. Scale bars: 1.0 cm (A) and 50 μm (B).

Modification of the functional properties of HDL is thought to be a viable approach to protect against atherosclerosis (38). With this in mind, we tested the effect of AMPK activation on atherosclerotic plaque formation in apoE^−/−^ mice. apoE^−/−^ mice fed a high-fat diet were treated with vehicle or AMPK activators for 10 weeks, and then whole dissected aortas and aortic valve sections stained with Oil red O were examined for lesions. Plaque formation was decreased by 41, 35, 38, and 45% in the entire aorta following A-769662, AICAR, metformin, and IMM-H007 treatment, respectively (**Fig. 7A, B**). Additionally, there were 43, 39, 48, and 54% reductions of plaque area in sections of aortic roots as a result of treatment with the indicated AMPK activators (Fig. 7C, D). Overall, these data indicate that AMPK activation by agonists with a range of mechanisms enhances HDL function and reduces atherosclerotic plaque formation.

## DISCUSSION

In the present study, we demonstrated a novel effect of AMPK activation and provided evidence that multiple molecular mechanisms underpin its atheroprotective effects. Our results provide the first evidence that activation of AMPK using a variety of substances enhances HDL function, including its beneficial effects on RCT, inflammation, and oxidative stress, all of which may contribute to a reduction in the formation of atherosclerotic lesions.

RCT is believed to be the primary mechanism by which HDL protects against atherosclerosis (39). There are three key stages in RCT. First, cholesterol is exported from macrophages to plasma HDL acceptors, mediated through macrophage ABCA1, ABCG1, and SR-BI. Second, free cholesterol in HDL is esterified by LCAT to form cholesteryl ester (CE) in the plasma, before being transferred directly to the liver using SR-BI or indirectly via CETP-mediated transfer to apoB-containing lipoproteins, and transported into hepatocytes using the LDL receptor. Finally, CE is excreted into the bile directly or after metabolism to form bile acids, and is either reabsorbed in the intestine or excreted in the feces.

Chemical activators of AMPK have frequently been used to investigate the role of AMPK activation in vitro and in vivo. Previously, it was shown that treatment with the AMP mimetic precursor, AICAR, enhances HDL-mediated cholesterol efflux from macrophages in vitro by increasing ABCG1 expression (31). A-769662 and salicylate activate AMPK by binding to the same site on the AMPKβ1 subunit (16, 40), and it was recently shown that both of these compounds are able to restore macrophage cholesterol homeostasis by stimulating cholesterol efflux through this mechanism. However, macrophage cholesterol efflux represents one component of the RCT process, and the effect of AMPK activation on the subsequent parts of the process has not been investigated in a suitable in vivo model of atherosclerosis. Here, we show for the first time that activation of AMPK through multiple mechanisms enhances the entire RCT process.

AMPK activation not only increased the export of cholesterol from peripheral macrophages to plasma, but also increased hepatic cholesterol uptake from plasma and the excretion of cholesterol in feces, all of which may explain the effect of AMPK activation on the reduction of atherosclerotic plaque formation. Regarding the first step of RCT, AMPK activation increased ABCA1 and ABCG1 expression without altering the expression of SR-BI and LXR in peripheral macrophages of mice, which is consistent with previous in vitro observations (12). As a result, in vivo plasma ^3^H-cholesterol levels were significantly higher in the AMPK activator-treated mice. In vitro AMPK activator-treated HDLs significantly enhanced ^3^H-cholesterol efflux from J774 macrophages. Furthermore, we also quantified the subsequent steps of RCT in the apoE^−/−^ mice. LCAT is critical for the maturation of HDL and plays a central role in RCT by facilitating transfer of excess cholesterol from peripheral tissues to the liver (41). SR-BI is the major hepatic receptor for HDL, where it mediates the selective uptake of CEs from HDL (42, 43). Our findings suggest that AMPK activation increases hepatic expression of both SR-BI and LCAT, implying that AMPK activation enhances the delivery of cholesterol from macrophages to the liver as the second step of RCT. Notably, it was found that the expression of LCAT mRNA and protein in the liver was increased significantly, stimulated by the indicated AMPK agonists, implicating that the activation of AMPK is helpful for the enhancement in the maturation of HDL in the second step of RCT. However, whether the activity of LCAT in the plasma was altered in the process warrants further investigation. Finally, we showed that AMPK activation increased the excretion of cholesterol into feces, supporting the notion that AMPK activation has positive effects on the entire process of RCT.

It is now well-accepted that atherosclerosis is a chronic inflammatory disease initiated by an accumulation and subsequent oxidation of LDL in the arterial wall (44). HDL contains several enzymes that have been linked to the antioxidant, anti-inflammatory, and lipid cargo-carrying functions of HDL (45). PON1, an enzyme located almost exclusively on HDL, has systemic antioxidant effects and promotes the atheroprotective properties of HDL. By contrast, MPO is a source of reactive oxygen species during inflammation and oxidizes apoA-I on HDL, thereby impairing its atheroprotective functions. Moreover, the ratio of MPO to PON1 expression is proposed to be a novel marker of HDL functionality and, therefore, to be useful in the prediction of coronary risk (32, 33). In the present study, we found that AMPK activation not only increased PON1 activity, but also decreased MPO activity, thus causing a reduction in MPO/PON1. In addition, the HII reflects the ability of HDL to prevent the formation of or to inactivate oxidized phospholipids. In the present study, HII was decreased by AMPK activation, further supporting the role of AMPK in improving the antioxidant and anti-inflammatory properties of HDL.

Macrophages play dynamic roles in all stages of atherosclerosis development, including uptake of retained lipoproteins, secretion of pro-inflammatory and anti-inflammatory cytokines, and regulation cholesterol homeostasis (46). Macrophages are phenotypically plastic cells and two types of macrophages have been identified: M1 (a pro-inflammatory phenotype) and M2 (an anti-inflammatory phenotype) (47). Growing evidence suggests that AMPK is involved in suppressing pro-inflammatory responses by modulating macrophage polarization (37). In the present study, AMPK activation polarized peritoneal macrophages to M2 phenotype in apoE^−/−^ mice, which was also characterized by higher ABCA1 expression, further supporting the role of AMPK in improving cholesterol homeostasis by HDL.

A recent study demonstrated that HDL may be modified and lose its atheroprotective effects during chronic inflammation or oxidative stress (48). Plasma levels of MDA, a product of lipid peroxidation and the pro-inflammatory cascade, are augmented by MCP-1, which recruits monocytes (49) and IL-6 (50), and all of these can modify HDL, leading to impairment of its atheroprotective effects (36). In the present study, plasma MDA, MCP-1, and IL-6 were reduced in mice treated with AMPK activators, suggesting that AMPK activation has antioxidant and anti-inflammatory effects in vivo, which likely contribute to the protection of HDL function.

In conclusion, this study provides the novel insight that pharmacological activation of AMPK can alleviate atherosclerosis by promoting HDL’s atheroprotective properties in apoE^−/−^ mice. These findings further support the potential of AMPK as a drug target for the treatment of atherosclerosis-related cardiovascular disease.
